# Supplementary material for: Netcombin: An algorithm for constructing optimal phylogenetic network from rooted triplets
Source: PLoS One. 2020 Sep 18;15(9):e0227842. doi: 10.1371/journal.pone.0227842 (PMC7500971; doi:10.1371/journal.pone.0227842)
Supplement: S4 File — (ZIP) [file pone.0227842.s004.zip › SequenceData-Triplet/sequenceData-10-20-30-0-Triplet/length 300, numseq 40, rate 10^-7/3 seq.pdf]

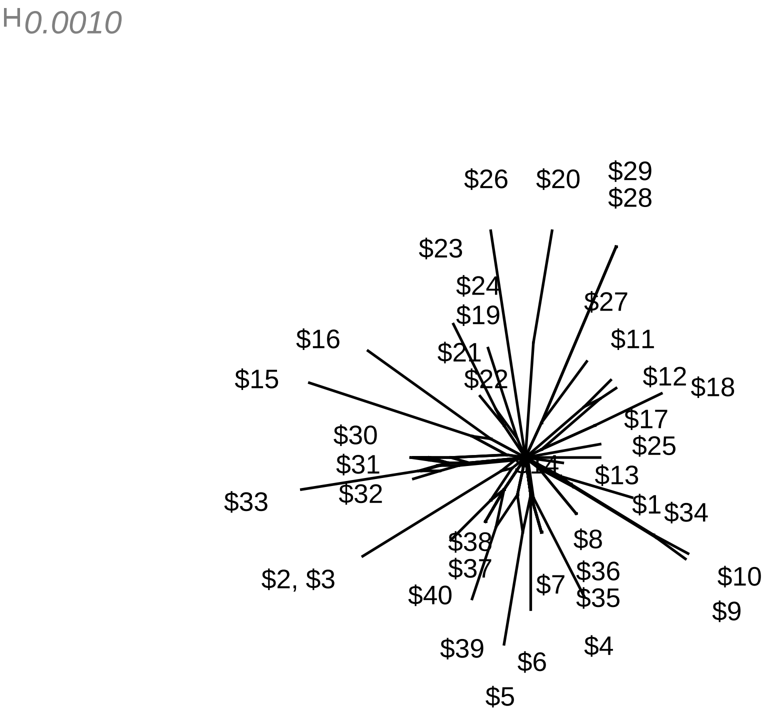
0.0010
$26
$20
$29
$28
$23
$24
$19
$27
$16
$11
$21
$15
$22
$12
$18
$17
$30
$25
$31
$14
$13
$32
$33
$1
$34
$38
$8
$37
$36
$2, $3
$10
$40
$7
$35
$9
$39
$4
$6
$5
